# Differences in clinical significance of bronchodilator responses measured by forced expiratory volume in 1 second and forced vital capacity

**DOI:** 10.1371/journal.pone.0282256

**Published:** 2023-02-24

**Authors:** Joon Young Choi, Sung Kyoung Kim, Jin Hwa Lee, Ki-Suck Jung, Kwang Ha Yoo, Ki-Eun Hwang, Jong Deog Lee, Yu-Il Kim, Hyoung Kyu Yoon, Soo-Jung Um

**Affiliations:** 1 Division of Pulmonary and Critical Care Medicine, Department of Internal Medicine, Incheon St. Mary’s Hospital, College of Medicine, The Catholic University of Korea, Seoul, Republic of Korea; 2 Division of Pulmonary and Critical Care Medicine, Department of Internal Medicine, St. Vincent’s Hospital, College of Medicine, The Catholic University of Korea, Seoul, Republic of Korea; 3 Department of Internal Medicine, Ewha Womans University Seoul Hospital, College of Medicine, Ewha Womans University, Seoul, Republic of Korea; 4 Division of Pulmonary, Allergy and Critical Care Medicine, Hallym University Sacred Heart Hospital, Hallym University Medical School, Anyang, Republic of Korea; 5 Division of Pulmonary, Allergy and Critical Care Medicine, Department of Internal Medicine, Konkuk University School of Medicine, Seoul, Republic of Korea; 6 Department of Internal Medicine, Institute of Wonkwang Medical Science, Wonkwang University, School of Medicine, Iksan, Jeonbuk, Republic of Korea; 7 Division of Pulmonary and Critical Care Medicine, Department of Internal Medicine, Gyeongsang National University, School of Medicine, Jinju, Republic of Korea; 8 Division of Pulmonary Medicine, Department of Internal Medicine, Chonnam National University Hospital, Gwangju, Republic of Korea; 9 Division of Pulmonology, Critical Care and Sleep Medicine, Department of Internal Medicine, Yeouido St. Mary’s Hospital, College of Medicine, The Catholic University of Korea, Seoul, Republic of Korea; 10 Department of Internal Medicine, Pulmonology Division, Dong-A University Hospital, College of Medicine, Dong-A University, Busan, Republic of Korea; Kaohsuing Medical University Hospital, TAIWAN

## Abstract

**Background:**

The clinical implication of bronchodilator response (BDR) is not fully understood. However, BDR is frequently present in patients with chronic obstructive pulmonary disease (COPD). We identified the differences in clinical features regarding BDR. In addition, we divided BDR into BDR for forced expiratory volume in 1 s (FEV1) and BDR for forced vital capacity (FVC; i.e., BDR-FEV1 and BDR-FVC, respectively) and analyzed clinical significance.

**Methods:**

We used data from the Korea COPD Subgroup Study, a multicenter cohort study of COPD patients recruited from 54 centers in South Korea since April 2012. We analyzed differences in baseline characteristics, 1-year exacerbation rate, and 3-year FEV1 decline between BDR negative and positive patients. Moreover, we analyzed the differences in clinical features between BDR-FEV1 positive and negative patients and between BDR-FVC positive and negative patients.

**Results:**

Of the 2,181 patients enrolled in this study, 366 (16.8%) were BDR positive. BDR positive patients were more likely to be ever-smokers and to have a lower body mass index and higher symptom scores compared to BDR negative patients. Baseline FEV1 and FEV1/FVC were lower in the BDR positive compared to the BDR negative group (1.7 ± 0.6 and 1.6 ± 0.5, respectively, p < 0.01; 50.9 ± 12.1 and 46.5 ± 14.8, respectively, p < 0.01). BDR positive patients were more likely to have been diagnosed with asthma–COPD overlap and to receive inhaled corticosteroids (ICS) than BDR negative patients. BDR-FVC patients were more likely to be smokers, suffer from worse symptoms and have lower lung function than those with no BDR-FVC. BDR had no significant effect on 1-year moderate to severe or severe exacerbation rates or 3-year annual FEV1 decline. Interactive effects of ICS and BDR on the exacerbation rate were not significant in any group.

**Conclusions:**

In this study, BDR positive patients were more likely to be ever-smokers and to have worse symptoms and lung function than BDR negative patients. BDR-FVC was associated with worse symptom control and lung function compared to BDR-FEV1. However, there were no significant differences in exacerbation rate or decline in lung function in any BDR group. In addition, the effects of ICS on exacerbations were not significant in any group.

## Introduction

Chronic obstructive pulmonary disease (COPD) is characterized by a fixed airway obstruction that persists after the administration of bronchodilators [1]. Bronchodilator response (BDR) is an important method for diagnosing airway reversibility, which is primarily observed in asthma but may also be observed in COPD [2–4]. It is significant in asthma because it reflects poor control of the disease; however, its clinical significance in COPD is not clear [5, 6]. Although several studies have shown that BDR is associated with a decline in lung function, exacerbation rate, and survival, few have shown a significant difference after adjustment for baseline lung function [7, 8].

BDR is defined as an increase in either the forced expiratory volume in 1 s (FEV1) or forced vital capacity (FVC) after the administration of a bronchodilator [9]. Recent studies have identified distinctive clinical features of FEV1 reversibility (BDR-FEV1) and FVC reversibility (BDR-FVC) [10–14]. BDR-FEV1 occurs more frequently in patients with mild airflow limitation, whereas BDR-FVC occurs more frequently in patients with severe disease, as assessed by the Global Initiative for Chronic Obstructive Lung Disease guidelines [15]. BDR-FVC is more common than BDR-FEV1 and is associated with severe symptoms and emphysema/small airway disease on chest computed tomography [10, 12]. However, few studies have evaluated the prospective trajectory of lung function or risk for exacerbation.

BDR is one of the most important features in diagnosing asthma–COPD overlap (ACO), similar to asthma [16]. However, recent studies suggest that positive BDR is not necessary to diagnose ACO because it is also frequently observed in COPD patients. Therefore, BDR may not predict responsiveness to inhaled corticosteroids (ICS) [17, 18]. In addition, BDR is highly variable, which limits its use as a diagnostic test for ACO [3, 18, 19]. Furthermore, it is unclear whether the therapeutic effects of ICS in COPD patients differ according to BDR.

In the present study, we investigated the clinical implication of BDR positivity in COPD patients. In addition, we analyzed clinical features according to two conventional BDR diagnostic criteria (i.e., BDR-FEV1 and BDR-FVC). The exacerbation rate was assessed with 1-year prospective data. In particular, we evaluated whether ICS use affects the risk for exacerbation. We analyzed 3-year annual lung function test results to evaluate differences in the FEV1 decline between BDR positive and negative patients.

## Materials and methods

### Study population and data collection

This study was based on data from the Korea COPD Subgroup Study (KOCOSS), a nationwide cohort study of COPD patients recruited from 54 medical centers in South Korea since April 2012 [20]. This cohort included patients age ≥40 years who had FEV1/FVC <0.7. Data were collected from electronic medical records of patients submitted by doctors or trained nurses. We extracted data from the KOCOSS database in November 2020.

### Definition of BDR

BDR was measured with two spirometry tests conducted 10–20 min after the administration of albuterol (200–400 μg) [21, 22]. In accordance with the American Thoracic Society (ATS)/European Respiratory Society (ERS) 2005 definition of BDR, we defined BDR as an increase in absolute volume of 200 mL and relative volume of 12% in either FEV1 or FVC after bronchodilator use compared to baseline values [9]. BDR-FEV1 and BDR-FVC indicated that the patients met the BDR criteria for FEV1 and FVC, respectively.

### Clinical parameters

We recorded baseline characteristics (including age, sex, smoking history, and body mass index [BMI]); medical history (history of asthma, ACO, and ICS use); baseline and 3-year annual lung function parameters (spirometry, lung volume, and the diffusing capacity of the lungs for carbon monoxide [DLco]); laboratory parameters of type 2 inflammation (blood eosinophil count, immunoglobulin E [IgE], and fractional exhaled nitric oxide [FeNO]); and scores on the modified Medical Research Council (mMRC) dyspnea scale, COPD assessment test (CAT), 6-min walk test (6MWT), and psychological tests (including the Beck Depression Inventory [BDI] for depression and the Beck Anxiety Inventory [BAI] for anxiety). Chest CT images were reviewed to identify patients with emphysema and bronchiectasis. Exacerbations that required the administration of antibiotics or oral corticosteroids were defined as moderate, and those that required an emergency room visit or hospitalization were defined as severe [1]. We recorded the numbers of total, moderate to severe, and severe exacerbations in a year.

### Ethics approval and consent to participate

Ethical approval was obtained from the Ethics Committee of each medical center participating in KOCOSS. Written informed consent was collected from all participating patients. All methods were carried out in accordance with relevant guidelines and regulations.

The names of ethics committees are shown below:

Gacheon University Gil Medical Center, Hallym University Kangnam Sacred Heart Hospital, Gangnam Severance Hospital, Kyung Hee University Hospital at Gangdong, Hallym University Kangdong Sacred Heart Hospital, Kangbuk Samsung Hospital, Kangwon National University Hospital, Konkuk University Hospital, Konkuk University Chungju Hospital, Kyungpook National University Hospital, Gyeongsang National University Hospital, Korea University Guro Hospital, Korea University Anam Hospital, Seoul Eulji Hospital, Dongguk University Gyeongju Hospital, Dongguk University Ilsan Hospital, Keimyung University Dongsan Medical Center, Dong-A University Hospital, Hallym University Dongtan Sacred Heart Hospital, Pusan National University Hospital, Inje University Busan Paik Hospital, The Catholic University of Korea Bucheon St Mary’s Hospital, Soonchunhyang University Hospital Bucheon, Seoul National University Bundang Hospital, Bundang CHA Hospital, Seoul Metropolitan Government Seoul National University Bora-mae Medical Center, Samsung Medical Center, Soonchunhyang University Hospital Seoul, The Catholic University of Korea Seoul St Mary’s Hospital, The Catholic University of Korea St Paul’s Hospital, The Catholic University of Korea St Vincent’s Hospital, Severance Hospital, Asan Medical Center, Ajou University Hospital, The Catholic University of Korea Yeouido St Mary’s Hospital, The Catholic University of Korea Uijeongbu St Mary’s Hospital, Yeungnam University Medical Center, Ulsan University Hospital, Wonkwang University Sanbon Hospital, Wonju Severance Christian Hospital, Ewha Womans University Mokding Hospital, Incheon St Mary’s Hospital, Inha University Hospital, Chonnam National University Hospital, Chonbuk National University Hospital, Jeju National University Hospital, Soonchunhyang University Hospital Cheonan, Hallym University Chuncheon Sacred Heart Hospital, Hallym University Sacred Heart Hospital, and Hanyang University Guri Hospital.

We also received approval from each center to use their subjects’ clinical records for the study while maintaining the confidentiality of the data. Written informed consent was collected from all participating patients.

### Statistical analyses

All statistical analyses were performed using R software (version 3.6.3; R Foundation for Statistical Computing, Vienna, Austria). Quantitative variables are presented as means ± standard deviations, and categorical variables are presented as frequencies (percentages). We analyzed differences in the clinical characteristics of BDR positive and negative patients. We also compared clinical characterisitcs of BDR-FEV1 positive and negative patients, and also BDR-FVC positive and negative patients. Differences in the categorical and continuous variables between two groups were analyzed using the χ^2^ test and Student’s *t* test, respectively. The frequency of exacerbation at the 1-year follow-up was analyzed with a negative binomial regression model. Regression models were adjusted for covariates, including age, sex, smoking status, baseline post-bronchodilator FEV1, and history of exacerbations in the 1 year preceding study enrollment. We performed subgroup analysis of exacerbation frequency according to different GOLD severity (GOLD I-II vs GOLD III-IV). In addition, the frequency of exacerbation and ICS use were compared between BDR positive and negative patients, BDR-FEV1 positive and negative patients, and BDR-FVC positive and negative patients, with negative regression models adjusted for identical covariates. We analyzed the annual FEV1 decline using a linear mixed model adjusted for covariates, including age, sex, smoking status, baseline post-bronchodilator FEV1, and history of exacerbations in the 1 year preceding study enrollment. We performed subgroup analysis of annual lung function decline according to different GOLD severity.

## Results

### Differences in baseline characteristics between BDR positive and negative patients

This study included 2,181 patients, including 366 (16.8%) BDR positive patients. Differences in the clinical characteristics of BDR positive and negative patients are shown in Table 1. There were no significant differences in age or sex distributions between the two groups. BDR positive patients were more likely to be ever-smokers and to have a lower BMI than BDR negative patients. Symptom scores, including scores on the mMRC dyspnea scale and CAT, were higher in the BDR positive compared to BDR negative group. However, 6MWT scores and psychological scores (BAI and BDI) were similar between the groups.

**Table 1:** Difference of clinical characteristics according to bronchodilator response.

	BDR (-) (n = 1815, 83.2%)	BDR (+) (n = 366, 16.8%)	P-value
Age	69.0 ± 7.7	68.6 ± 7.8	0.32
Sex (male)	1682 (92.7%)	348 (95.1%)	0.12
Smoking Hx			0.04
• Never	142 (7.9%)	17 (4.7%)	
• Ever-smoker	1660 (92.1%)	346 (95.3%)	
BMI	23.1 ± 3.5	22.6 ± 3.1	<0.01
mMRC	1.3 ± 0.9	1.4 ± 0.9	0.02
CAT score	14.2 ± 7.9	15.7 ± 8.5	<0.01
6MWT	383.4 ± 114.1	385.6 ± 124.6	0.78
BDI score	6.9 ± 8.2	6.7 ± 8.1	0.79
BAI score	4.4 ± 6.6	4.4 ± 6.3	0.94
GOLD stage			<0.01
• I	205 (11.3%)	8 (2.2%)	
• II	982 (54.1%)	144 (39.3%)	
• III	503 (27.7%)	166 (45.4%)	
• IV	124 (6.8%)	48 (13.1%)	
postBD FEV1 (L)	1.7 ± 0.6	1.6 ± 0.5	<0.01
postBD FVC (L)	3.3 ± 0.8	3.4 ± 0.8	<0.01
FEV1/FVC	50.9 ± 12.1	46.5 ± 14.8	<0.01
FEF_25-75_	28.9 ± 15.0	24.8 ± 13.1	<0.01
DLCO	64.0 ± 20.7	63.6 ± 20.8	0.76
RV/TLC	0.4 ± 0.1	0.5 ± 0.1	<0.01
Asthma Hx.	514 (28.7%)	116 (32.2%)	0.20
ACO	185 (18.8%)	78 (39.4%)	<0.01
Eosinophil count	224.7 ± 254.8	233.0 ± 212.8	0.55
IgE	232.1 ± 349.7	243.9 ± 374.5	0.72
FENO	26.8 ± 16.4	29.5 ± 20.6	0.44
ICS use	615 (36.8%)	152 (44.4%)	0.01
Emphysema	402 (43.8%)	101 (50.8%)	0.09
Bronchiectasis	110 (12.0%)	23 (11.6%)	0.70
M-S exacerbation (Y/N)	528 (40.2%)	116 (42.3%)	0.55
M-S exacerbation (Frequency)	1.1 ± 2.0	1.1 ± 2.2	0.57
S exacerbation (Y/N)	140 (10.7%)	29 (10.6%)	1.00
S exacerbation (Frequency)	0.2 ± 0.7	0.1 ± 0.5	0.32

BDR, bronchodilator response; BMI, body mass index; mMRC, modified Medical Research Council; CAT score, COPD Assessment Test; 6MWT, 6-minute walking test; BDI, Beck depression inventory; BAI, Beck anxiety inventory; GOLD, global initiative for chronic obstructive lung disease; FEV1, forced expiratory volume in 1 second; FVC, forced vital capacity; FEF_25-75,_ Forced expiratory flow between 25% and 75%; RV, residual volume; TLC, total lung capacity; ACO, asthma-COPD overlap; IgE, immunoglobulin E; FENO, fractional exhaled nitric oxide; ICS, inhaled corticosteroid; M-S exacerbation, moderate-to-severe exacerbation; S exacerbation, severe exacerbation

There were no differences in asthma history, blood eosinophil count, or IgE and FeNO between the groups. However, ACO had been diagnosed more frequently in the BDR positive group compared to the BDR negative group (39.4% and 18.8%, respectively, p < 0.01). In addition, ICS were prescribed more frequently in the BDR positive group compared to the BDR negative group (44.4% and 36.8%, respectively, p < 0.01). There were no significant differences between the groups in the numbers of patients with emphysema or bronchiectasis on chest CT.

### Differences in baseline characteristics between BDR-FEV1 positive vs negative, and BDR-FVC positive vs negative patients

Of the 2,181 patients in this study, 240 (11.0%) were BDR-FEV1 positive and 211 (9.7%) were BDR-FVC positive. BDR-FEV1 positive patients were younger than BDR negative and BDR-FVC patients. BDR-FVC positive patients, but not BDR-FEV1 patients, were more likely to be ever-smokers and had a lower BMI than the BDR negative group. Respiratory symptom scores, including scores on the mMRC dyspnea scale and CAT, were worse for BDR-FVC positive patients compared to BDR negative patients. The 6MWT and psychological scores did not differ significantly among the BDR-FEV1, BDR-FVC, and BDR negative groups.

The number of patients with asthma was similar between the BDR-FEV1 and BDR negative groups but was higher for BDR-FVC positive patients compared to BDR negative patients. There were no significant differences in blood eosinophil counts or IgE and FeNO between the BDR positive and negative groups. However, ACO had been diagnosed more frequently in the BDR-FEV1 positive and BDR-FVC positive groups compared to the BDR negative group. Thus, both BDR positive groups received more ICS than the BDR negative group. There were no significant differences between the groups in the number of patients with emphysema or bronchiectasis on chest CT.

### Exacerbations

**Fig 1:**
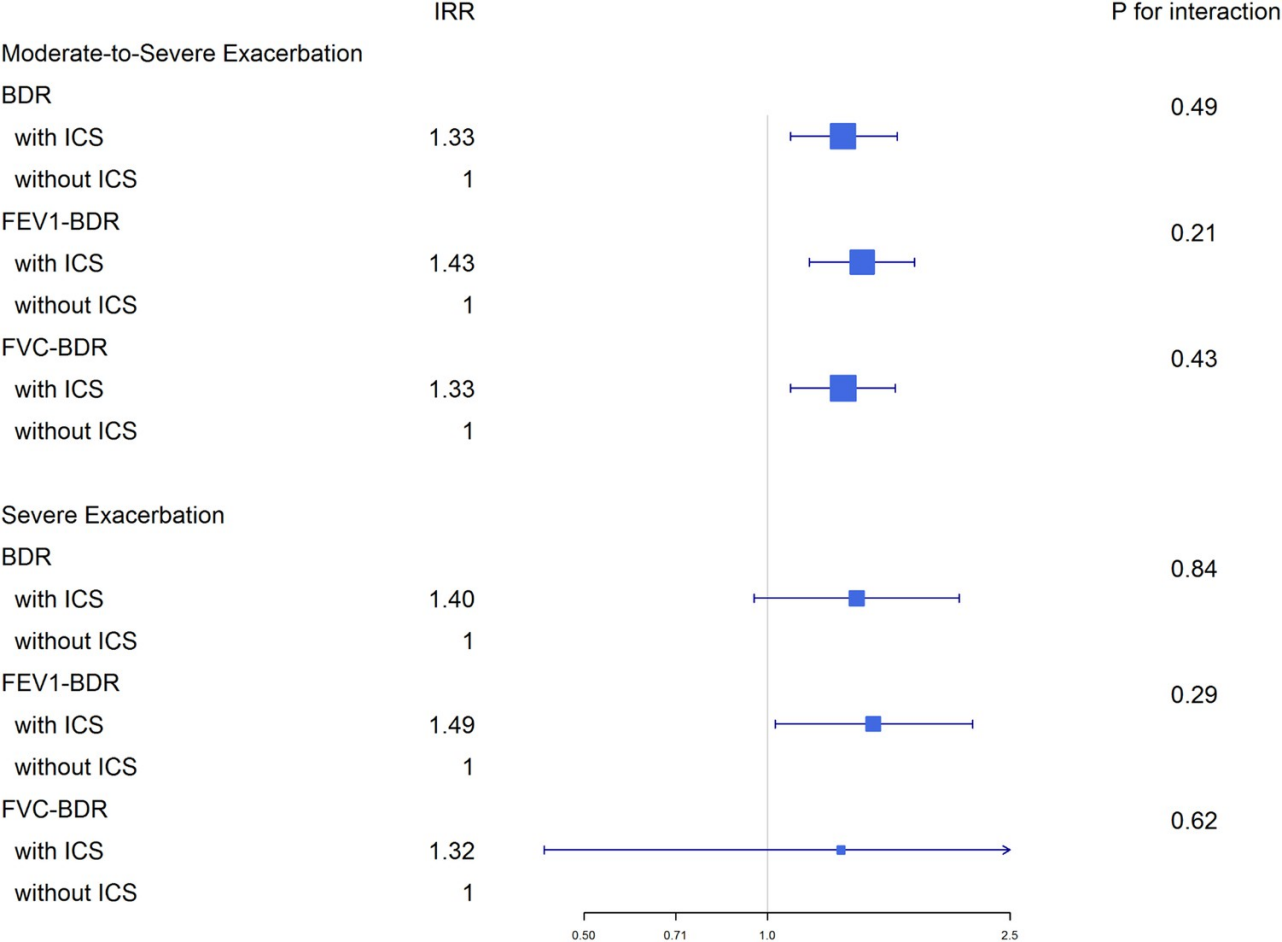
Interactive effects of ICS and BDR on the exacerbation rate.

In the 1 year prior to study enrollment, there were no significant differences in the numbers of moderate to severe or severe exacerbations between the BDR positive and negative groups (Table 1). A greater proportion of BDR-FVC positive patients experienced moderate to severe exacerbations compared to BDR-FVC negative patients, but the frequency of exacerbations was not different between the groups (Table 2). Therefore, BDR-FEV1 positive patients experienced more frequent severe exacerbations compared to BDR-FEV1 negative patients. The frequency of exacerbations in the 1 year follow-up after study enrollment according to BDR status is shown in Table 3. Irrespective of BDR status, BDR positivity did not affect the frequency of moderate to severe or severe exacerbations. In subgroup analysis stratified by disease severity, BDR positive patients had less frequent severe exacerbation compared to BDR negative patients in GOLD III-IV patients (IRR = 0.48, p<0.01) (S2 Table in S1 File). However, positivity of BDR-FEV1 or BDR-FVC did not show statistical significance on frequency of exacerbation. Also, interactive effects on disease severity and BDR or BDR-FVC on severe exacerbation rate were significant; however BDR-FEV1 showed no interactive effects (S1 Fig). Furthermore, interactive effects of ICS and BDR on the exacerbation rate were not significant in any group (Fig 1).

**Table 2:** Differences of clinical characteristics according to bronchodilator response defined by FEV1 response and FVC response.

	BDR-FEV1 (-) (n = 1941, 89.0%)	BDR-FEV1 (+) (n = 240, 11.0%)	P-value	BDR-FVC (-) (n = 1970, 90.3%)	BDR-FVC (+) (n = 211, 9.7%)	P-value
Age	69.1 ± 7.7	67.7 ± 7.8	<0.01	68.9 ± 7.8	69.6 ± 7.5	0.20
Sex (male)	1802 (92.8%)	228 (95.0%)	0.27	1827 (92.7%)	203 (96.2%)	0.08
Smoking Hx			0.12			0.03
• Never	148 (7.7%)	11 (4.6%)		152 (7.8%)	7 (3.3%)	
• Ever-smoker	1779 (92.3%)	227 (95.4%)		1804 (92.2%)	202 (96.7%)	
BMI	23.0 ± 3.5	22.8 ± 3.0	0.25	23.0 ± 3.5	22.6 ± 3.1	0.06
mMRC	1.31 ± 0.9	1.36 ± 0.9	0.50	1.30 ± 0.9	1.51 ± 0.9	<0.01
CAT score	14.4 ± 8.0	15.0 ± 8.3	0.28	14.3 ± 8.0	16.3 ± 8.3	<0.01
6MWT	382.5 ± 115.6	394.2 ± 117.4	0.22	385.2 ± 114.5	370.0 ± 127.9	0.12
BDI score	6.9 ± 8.2	6.5 ± 7.9	0.63	6.9 ± 8.1	6.8 ± 8.6	0.96
BAI score	4.4 ± 6.6	4.3 ± 6.0	0.80	4.3 ± 6.5	5.2 ± 7.5	0.27
GOLD stage			<0.01			<0.01
• I	210 (10.8%)	3 (1.2%)		208 (10.6%)	2 (2.4%)	
• II	1019 (52.5%)	107 (44.6%)		1071 (54.4%)	55 (26.1%)	
• III	559 (28.8%)	110 (45.8%)		561 (28.5%)	108 (51.2%)	
• IV	152 (7.8%)	20 (8.3%)		129 (6.6%)	43 (20.4%)	
postBD FEV1 (L)	1.7 ± 0.6	1.8 ± 0.5	0.01	1.7 ± 0.6	1.4 ± 0.5	<0.01
postBD FVC (L)	3.3 ± 0.8	3.6 ± 0.8	<0.01	3.3 ± 0.8	3.2 ± 0.8	0.22
FEV1/FVC	50.7 ± 12.9	45.2 ± 9.5	<0.01	50.5 ± 12.0	47.0 ± 17.7	<0.01
FEF_25-75_	28.3 ± 15.0	26.9 ± 13.1	0.14	28.9 ± `4.9	21.2 ± 10.8	<0.01
DLCO	63.7 ± 20.7	66.1 ± 20.6	0.11	64.3 ± 20.7	60.9 ± 20.6	0.04
RV/TLC	0.4 ± 0.1	0.4 ± 0.1	0.09	0.4 ± 0.1	0.5 ± 0.1	<0.01
Asthma Hx.	558 (29.1%)	72 (30.6%)	0.69	558 (28.7%)	72 (34.6%)	<0.01
ACO	201 (19.2%)	62 (45.6%)	<0.01	227 (21.1%)	36 (34.6%)	<0.01
Eosinophil count	223.1 ± 250.3	250.6 ± 230.0	0.14	227.7 ± 253.0	211.2 ± 197.8	0.32
IgE	231.8 ± 343.8	252.1 ± 431.6	0.66	235.6 ± 363.0	211.9 ± 196.4	0.37
FENO	26.8 ± 16.4	30.3 ± 21.9	0.48	26.8 ± 16.5	31.5 ± 23.4	0.49
ICS use	667 (37.3%)	100 (44.4%)	0.046	676 (37.2%)	91 (46.4%)	0.02
Emphysema	442 (44.6%)	61 (48.8%)	0.42	443 (44.3%)	60 (51.3%)	0.18
Bronchiectasis	118 (11.9%)	15 (12.0%)	1.00	118 (11.8%)	15 (12.8%)	0.87
M-S exacerbation (Y/N)	581 (41.1%)	63 (36.2%)	0.25	566 (39.6%)	78 (49.1%)	0.03
M-S exacerbation (Frequency)	1.1 ± 2.0	1.0 ± 2.0	0.45	1.0 ± 2.0	1.4 ± 2.5	0.10
S exacerbation (Y/N)	155 (11.0%)	14 (8.0%)	0.30	147 (10.3%)	22 (13.8%)	0.22
S exacerbation (Frequency)	0.2 ± 0.7	0.1 ± 0.4	<0.01	0.2 ± 0.7	0.2 ± 0.6	0.63

BDR, bronchodilator response; BMI, body mass index; mMRC, modified Medical Research Council; CAT score, COPD Assessment Test; 6MWT, 6-minute walking test; BDI, Beck depression inventory; BAI, Beck anxiety inventory; GOLD, global initiative for chronic obstructive lung disease; FEV1, forced expiratory volume in 1 second; FVC, forced vital capacity; FEF_25-75,_ Forced expiratory flow between 25% and 75%; RV, residual volume; TLC, total lung capacity; ACO, asthma-COPD overlap; IgE, immunoglobulin E; FENO, fractional exhaled nitric oxide; ICS, inhaled corticosteroid; M-S exacerbation, moderate-to-severe exacerbation; S exacerbation, severe exacerbation

**Table 3:** Risk of exacerbation according to BDR.

	IRR	95%CI	p-value
Moderate-to-severe exacerbation			
BDR	1.05	0.84–1.32	0.65
BDR-FEV1	1.02	0.77–1.35	0.91
BDR-FVC	1.08	0.82–1.43	0.60
Severe exacerbation			
BDR	0.78	0.49–1.23	0.29
BDR-FEV1	0.66	0.34–1.21	0.20
BDR-FVC	0.85	0.50–1.44	0.54

* adjusted by age, sex, smoking status, post-bronchodilator FEV1 and previous exacerbation history preceding 1-year of enrollment.

IRR, incidence rate ratio; BDR, bronchodilator response; FEV1, forced expiratory volume in 1 second; FVC, forced vital capacity

### Pulmonary function

**Fig 2:**
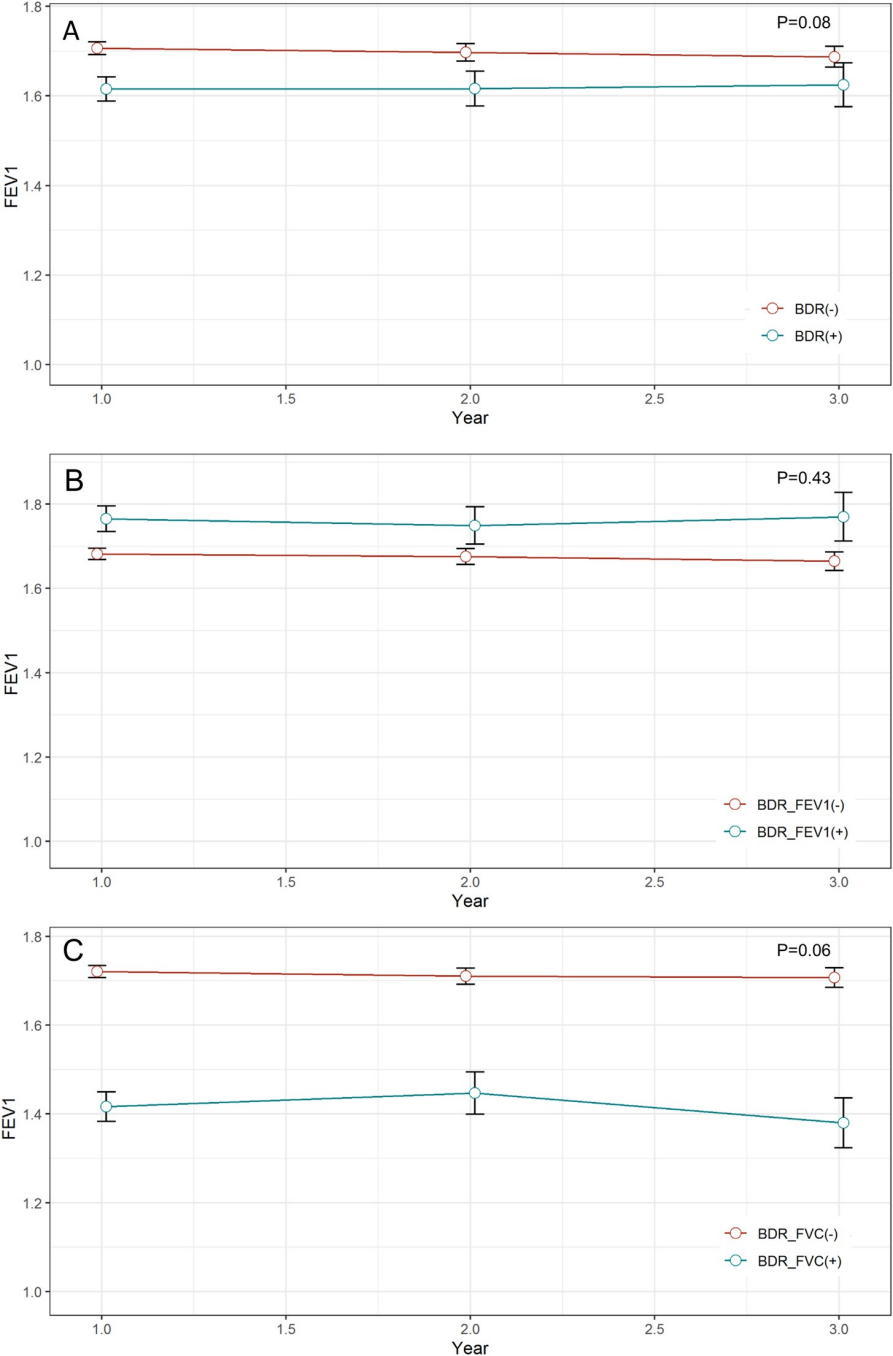
Difference of lung function trajectories according to (A) BDR, (B) BDR-FEV1, and (C) BDR-FVC.

Post-bronchodilator FEV1 was lower in BDR positive patients compared to BDR negative patients (1.6 ± 0.5 and 1.7 ± 0.6, respectively, p < 0.01). Thus, a greater number of patients in the BDR positive group compared to the BDR negative group were classified as GOLD stage III–IV (58.5% and 34.5%, respectively, p < 0.01; Table 1). FEV1/FVC was lower and residual volume per total lung capacity was higher in the BDR positive group compared to the BDR negative group. Post-bronchodilator FEV1 was higher in the BDR-FEV1 positive group compared to the BDR-FEV1 negative group but lower in the BDR-FVC positive group compared to the BDR-FVC negative group (Table 2). FEV1/FVC was lower in the BDR-FEV1 and BDR-FVC positive groups compared to the BDR negative group. Furthermore, compared to the BDR negative group, DLco was lower and RV/TLC was higher in the BDR-FVC positive group but not the BDR-FEV1 group. Prospective analyses of 3-year lung function tests showed no significant difference in annual decline rate in absolute volume of FEV1 according to BDR positivity in any group (Fig 2).

## Discussion

We explored the clinical features of COPD patients according to BDR positivity, as defined by the ATS/ERS definition. We divided COPD patients into those with FEV1 responsiveness (i.e., BDR-FEV1) and those with FVC responsiveness (i.e., BDR-FVC), then compared the clinical characteristics of these patients with BDR-FEV1 and BDR-FVC negative patients, respectively. BDR positive patients were more likely to be ever-smokers and to have severe symptoms and worse lung function compared to BDR negative patients. BDR positive patients had been more frequently diagnosed with ACO and prescribed ICS compared to BDR negative patients. However, Th2 inflammatory biomarkers did not differ significantly between the groups. In addition, there were no significant differences in the risk for exacerbation or decline in lung function according to BDR, BDR-FEV1, or BDR-FVC.

BDR positive COPD patients have lower FEV1 and FEV1/FVC than BDR negative patients [23–25]. One possible explanation for the result is that BDR positive group composes more of ACO patients compared to BDR negative patients; ACO is widely reported to be associated with poor lung function [26].However, the results differ between BDR-FEV1 and BDR-FVC patients. Although BDR-FVC positive patients have lower FEV1 compared to BDR-FVC negative patients, BDR-FEV1 positive patients may have better [10] or worse [13] lung function than BDR-FEV1 negative patients. Our study found lower FEV1 in BDR-FVC positive patients and higher FEV1 in BDR-FEV1 positive patients. This is consistent with previous results that showed that BDR-FEV1 positive patients more often had mild to moderate COPD, whereas BDR-FVC positive patients more often had severe COPD [15]. This may be because FEV1 is mainly affected by airflow limitation at high lung volumes, whereas FVC is mainly affected by airflow at low lung volumes [7, 15]. Numerous studies have shown that BDR positive patients have a rapid decline in lung function [27, 28]. However, the difference between BDR positive and negative patients is not statistically significant after baseline FEV1 is adjusted for [8, 17, 29]. Our study found similar results: After we adjusted for baseline lung function, there were no significant differences in 3-year changes in FEV1 between the BDR groups.

Although BDR is associated with worse control of disease and frequent use of reliever medications in asthmatic patients [5, 6], it is not associated with the risk for COPD exacerbations [7, 8, 10, 11, 13]. In the ECLIPSE study, although the exacerbation rate was significantly higher in patients with less reversible than more reversible airway restriction, the results may have been affected by the lack of adjustment for baseline FEV1 [8, 28]. Recently, ERS/ATS released new technical standard on interpretive strategies for lung function tests, which newly defined BDR as an at least 10% increase in either FEV1%predicted or FVC %predicted [30]. It was based on epidemiologic data of health adult reported upper limit (95% percentile), and excluding absolute volume on diagnostic criteria to avoid errors for those with lower baseline lung function. Bhatt et al. analyzed COPDGene cohort to investigate clinical significance of new BDR definition on COPD patients, and showed that BDR positivity was also not associated with exacerbation rates or survival rates [31]. In our study, we showed that BDR positive patients showed lesser frequent severe exacerbation compared to BDR negative patients in GOLD III-IV group. Interactive effects with disease severity on severe exacerbation risk were only significant in BDR and BDR-FVC, not in BDR-FEV1. Further investigations of BDR positivity and exacerbation risk in patients with lower lung function are needed.

By contrast, many studies have shown that BDR is associated with poor control of COPD symptoms, similar to asthma [12, 27, 32, 33]. However, some studies have reported negative results [13]. It is important to note that BDR-FEV1 is associated with wheezing and BDR-FVC with breathlessness [32, 33]. In our study, BDR and BDR-FVC was associated with high mMRC and CAT scores, which may be because BDR and BDR-FVC were more frequently present at higher GOLD stages compared to BDR-FEV1. Also, BDR-FVC are reported to be associated with lung hyperinflation and gas trapping causing more dyspnea symptom and lower exercise capacity [11, 33].

ACO is characterized by overlapping clinical manifestations of both COPD and asthma. Identifying ACO in COPD patients is important because such patients benefit from the use of ICS [1]. Although there is no consensus on the definition of ACO, most studies include BDR in the diagnostic criteria [16]. However, in Jo *et al*.’s analysis of several factors, including history of asthma, history of atopy, history of allergic rhinitis, BDR, and blood eosinophil and IgE, only blood eosinophil count (≥300 cell/μL) was associated with a positive response of exacerbations to ICS [34]. Our study showed similar results: The interaction of BDR with ICS use did not predict exacerbation risk. Furthermore, there were no differences in Th2 inflammatory markers (e.g., blood eosinophil count or IgE and FeNO) between BDR positive and negative patients. BDR may also be present in COPD patients without asthma [4, 35], which suggests that BDR may not be appropriate for diagnosing ACO. In the present study, BDR positive patients were often diagnosed with ACO and prescribed ICS; therefore, other factors, in particular blood eosinophil count, should be considered when deciding whether to prescribe ICS.

Although BDR positive patients have distinct features, such as poor symptom control and lung function, there are several limitations to considering BDR a phenotype. Most important is that BDR varies significantly over time, and reversibility decreases as the disease progresses [3, 19]. Moreover, previous studies have shown that BDR varies with baseline lung function. Patients with lower FEV1 may easily meet the BDR percentage criterion but are unlikely to meet the BDR absolute volume criterion [9]. In addition, patients with poor lung function have a low probability of having BDR [7, 10, 17].

Our study has several limitations. First, this study included patients recruited since 2012, and those enrolled before 2016 were prescribed ICS based on a history of frequent exacerbations and poor lung function, as recommended by the previous GOLD guidelines. This may have introduced bias into the study results, because patients with frequent exacerbations were more likely to have received ICS at baseline, which makes the analyses of the effectiveness of ICS unreliable. To overcome this limitation, we included history of exacerbations as a covariate in the regression model. Second, because the KOCOSS cohort includes patients that presented to tertiary hospitals, the study participants may be representative of the general COPD population and sufficient patients with mild COPD may not have been included. Third, we collected ICS prescription data at the baseline, and the adherence of the drug may not be adequate in the study period. Forth, there may be significant overlap between BDR-FEV1 and BDR-FVC. As result, comparison of clinical characteristics between BDR with either definition may have shown similar results. Finally, as shown in Fig 2, lung function trajectory of 3-year follow-up showed relatively low rate of lung function decline. The possible explanation for the gradual gradient is that the patients initially enrolled in this study may have been more aware of the disease and used appropriate drugs with higher adherence.

Despite these limitations, our study has several strengths. First, the study included a large number of patients enrolled in a nationwide multicenter cohort study for 8 years. We performed comprehensive analyses of the clinical characteristics of patients with BDR, BDR-FEV1, and BDR-FVC. We also performed prospective analyses of disease exacerbations and decline in lung function, which are the most important clinical parameters of COPD. Second, although it is frequently done in clinical practice, diagnosing ACO and ICS use according to BDR is unreliable. To the best of our knowledge, few studies have reported results similar to ours; the results of these studies are important for establishing diagnostic criteria for ACO and developing treatment guidelines.

## Conclusion

We analyzed the clinical characteristics of BDR positive and negative COPD patients. BDR positive patients had more severe symptoms and worse lung function compared to BDR negative patients. However, no significant differences were observed in the number of exacerbations or decline in lung function. In addition, the effects of ICS on disease exacerbations according to BDR were not significant. ACO had been diagnosed more frequently and ICS was used more frequently in BDR positive than BDR negative patients; therefore, the current treatment strategy should be reconsidered. We separately analyzed the clinical features of BDR-FEV1 and BDR-FVC positive patients. BDR-FVC positive patients had worse symptom control and lung function compared to BDR-FEV1 positive patients. BDR-FEV1 and BDR-FVC positivity did not affect the exacerbation rate or FEV1 decline.

## Supporting information

S1 Fig Interactive effects of disease severity and BDR on the exacerbation rate. (TIF)

S1 File (DOCX)
