# Supplementary figures and images for: Differences in clinical significance of bronchodilator responses measured by forced expiratory volume in 1 second and forced vital capacity
Source: PLoS One. 2023 Feb 24;18(2):e0282256. doi: 10.1371/journal.pone.0282256 (PMC9955608; doi:10.1371/journal.pone.0282256)

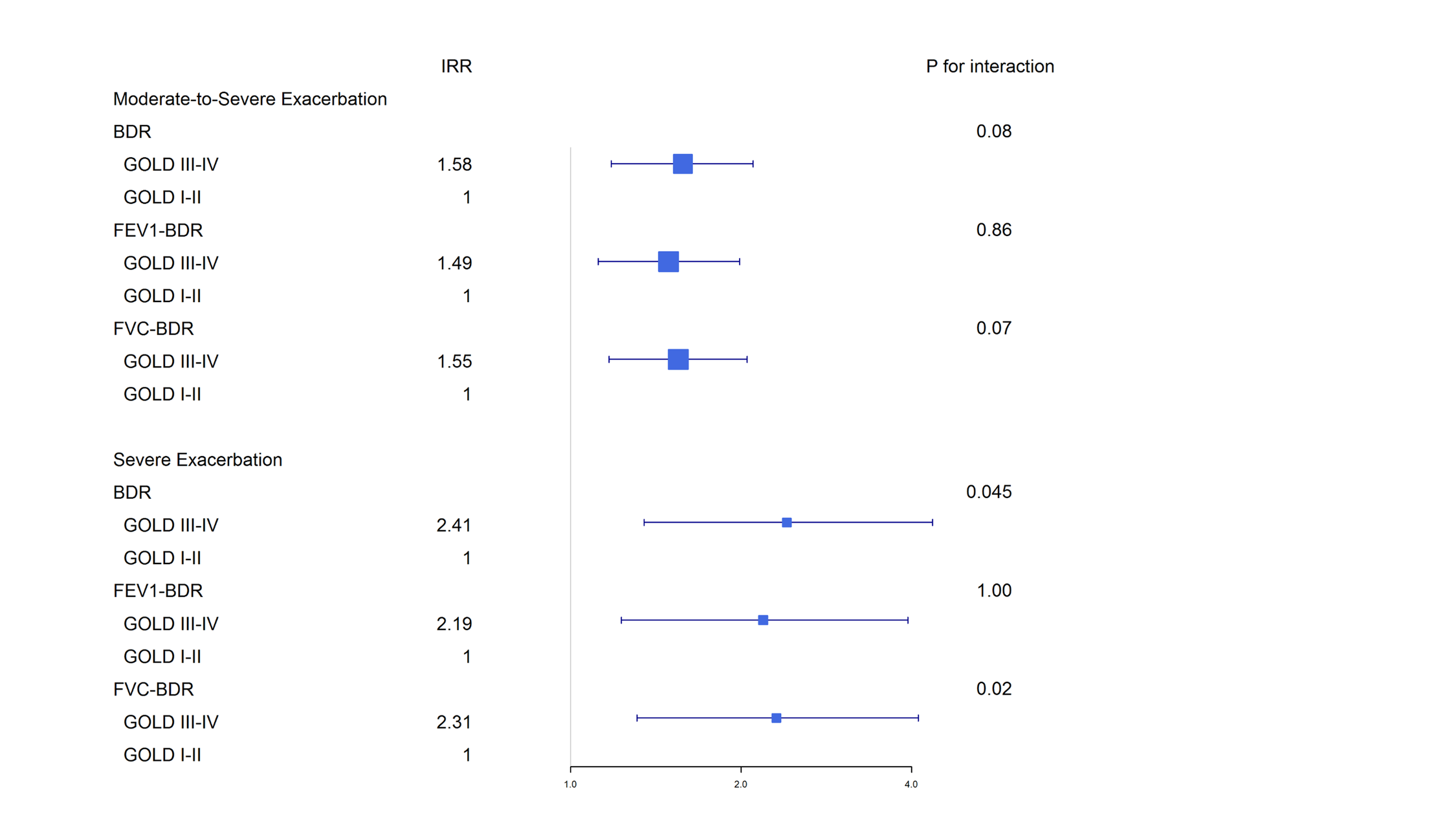

Supplement: S1 Fig — (TIF) [file pone.0282256.s001.tif]
