# Supplementary material for: Differences in clinical significance of bronchodilator responses measured by forced expiratory volume in 1 second and forced vital capacity
Source: PLoS One. 2023 Feb 24;18(2):e0282256. doi: 10.1371/journal.pone.0282256 (PMC9955608; doi:10.1371/journal.pone.0282256)

**Supplementary material**

Table S1. Result of multivariable linear mixed model that analyze FEV1 decline over time according to three different BDR definitions

|  | β-coefficient | p-value | |
| --- | --- | --- | --- |
| BDR | -22.6 | | 0.22 |
| Time | 0.9 | | 0.82 |
| BDR & Time | 17.1 | | 0.08 |
| BDR-FEV1 | -10.6 | | 0.63 |
| Time | 2.8 | | 0.48 |
| BDR-FEV1 & Time | 9.2 | | 0.43 |
| BDR-FVC | -35.0 | | 0.13 |
| Time | 1.6 | | 0.68 |
| BDR-FVC & Time | 23.7 | | 0.61 |

Table S2. Subgroup analysis of exacerbation frequency according to different COPD severity.

|  | IRR | 95%CI | p-value | IRR | 95%CI | p-value |
| --- | --- | --- | --- | --- | --- | --- |
| GOLD I-II | | | | GOLD III-IV | | |
| Moderate-to-severe exacerbation | | | |  |  |  |
| BDR | 1.19 | 0.79-1.80 | 0.42 | 0.77 | 0.59-1.00 | 0.052 |
| BDR-FEV1 | 0.97 | 0.59-1.63 | 0.92 | 1.01 | 0.72-1.45 | 0.94 |
| BDR-FVC | 1.60 | 0.89-3.00 | 0.13 | 0.42 | 0.66-1.20 | 0.88 |
| Severe exacerbation | | | |  |  |  |
| BDR | 1.29 | 0.49-3.26 | 0.60 | 0.48 | 0.28-0.79 | <0.01 |
| BDR-FEV1 | 0.53 | 0.08-2.19 | 0.41 | 0.64 | 0.31-1.29 | 0.23 |
| BDR-FVC | 2.50 | 0.75-8.45 | 0.14 | 0.60 | 0.33-1.06 | 0.08 |

Table S3. Subgroup analysis of FEV1 decline over time according to three different BDR definition

|  | β-coefficient | p-value |
| --- | --- | --- |
| GOLD I-II |  |  |
| BDR | -17.9 | 0.49 |
| Time | -16.2 | <0.01 |
| BDR & Time | 14.8 | 0.30 |
| BDR-FEV1 | 3.3 | 0.91 |
| Time | -14.0 | <0.01 |
| BDR-FEV1 & Time | -7.2 | 0.67 |
| BDR-FVC | -19.4 | 0.63 |
| Time | -15.0 | <0.01 |
| BDR-FVC & Time | 10.9 | 0.63 |
| GOLD III-IV |  |  |
| BDR | 6.7 | 0.80 |
| Time | 32.0 | <0.01 |
| BDR & Time | -0.1 | 0.99 |
| BDR-FEV1 | 7.0 | 0.83 |
| Time | 30.9 | <0.01 |
| BDR-FEV1 & Time | 5.9 | 0.72 |
| BDR-FVC | -1.0 | 0.97 |
| Time | 31.1 | <0.01 |
| BDR-FVC & Time | 5.3 | 0.75 |

Table S4. Differences of clinical characteristics between BDR-FEV1 (+) & BDR-FVC (+), BDR-FEV1 (+) & BDR-FVC (-) and BDR-FEV1(-) & BDR-FVC (+) groups.

|  | BDR-FEV1 (+) & BDR-FVC (+)  (n=84, 23.2%) | BDR-FEV1 (+) & BDR-FVC (-)  (n=154, 42.5%) | BDR-FEV1 (-) & BDR-FVC (+)  (n=124, 34.3%) | P-value |
| --- | --- | --- | --- | --- |
| Age | 68.6±7.6 | 67.2±7.9 | 70.1±7.4 | 0.01 |
| Sex (male) | 82 (97.6%) | 144 (93.5%) | 119 (96.0%) | 0.33 |
| Smoking Hx |  |  |  | 0.32 |
| -Never | 1 (1.2%) | 10 (6.5%) | 6 (4.9%) |  |
| -Ever-smoker | 82 (98.8%) | 143 (93.5%) | 117 (95.1%) |  |
| BMI | 23.1±2.9 | 144 (93.5%) | 22.2±3.3 | 0.12 |
| mMRC | 1.44±0.85 | 1.30±0.88 | 1.53±0.92 | 0.33 |
| CAT score | 15.4±7.8 | 14.8±8.7 | 16.8±8.7 | 0.14 |
| 6MWT | 369.2±116.0 | 408.1±117.3 | 371.8±136.4 | 0.06 |
| BDI score | 6.5±8.5 | 6.6±7.5 | 7.1±8.8 | 0.90 |
| BAI score | 5.6±8.3 | 3.7±4.7 | 5.0±7.0 | 0.31 |
| GOLD stage |  |  |  | <0.01 |
| - I | 0 (0.0%) | 3 (1.9%) | 5 (4.0%) |  |
| - II | 18 (21.4%) | 89 (57.8%) | 37 (29.8%) |  |
| - III | 51 (60.7%) | 57 (37.0%) | 55 (44.4%) |  |
| - IV | 15 (17.9%) | 5 (3.2%) | 27 (21.8%) |  |
| postBD FEV1 (L) | 1.5±0.4 | 1.9±0.5 | 1.3±0.5 | <0.01 |
| postBD FVC (L) | 3.4±0.8 | 3.7±0.8 | 3.1±0.8 | <0.01 |
| FEV1/FVC | 44.0±9.3 | 46.1±9.5 | 49.2±21.6 | 0.04 |
| FEF_25-75_ | 22.0±8.9 | 29.7±14.2 | 20.7±11.9 | <0.01 |
| DLCO | 64.3±20.8 | 67.0±20.7 | 58.9±20.5 | 0.01 |
| RV/TLC | 0.5±0.1 | 0.4±0.1 | 0.5±0.1 | <0.01 |
| Asthma Hx. | 28 (34.1%) | 44 (29.1%) | 44 (35.8%) | 0.48 |
| ACO | 20 (47.6%) | 42 (44.7%) | 6 (25.8%) | 0.03 |
| Eosinophil count | 229.8±233.6 | 262.9±229.6 | 194.7±169.1 | 0.053 |
| IgE | 189.4±182. | 8277.8±498. | 0226.1±205.3 | 0.55 |
| FENO | 35.9±28.7 | 27.6±18.2 | 26.5±16.4 | 0.65 |
| ICS use | 38 (48.7%) | 60 (41.4%) | 50 (43.5%) | 0.57 |
| Emphysema | 20 (46.5%) | 40 (49.4%) | 39 (53.4%) | 0.76 |
| Bronchiectasis | 7 (16.3%) | 8 (9.9%) | 8 (11.0%) | 0.56 |
| M-S exacerbation (Y/N) | 25 (43.1%) | 38 (33.3%) | 51 (52.0%) | 0.02 |
| M-S exacerbation  (Frequency) | 1.3±2.6 | 0.8±1.6 | 1.4±2.5 | 0.10 |
| S exacerbation (Y/N) | 7 (12.1%) | 7 (6.1%) | 15 (15.3%) | 0.09 |
| S exacerbation (Frequency) | 1.3 ± 2.6 | 0.8 ± 1.6 | 1.4 ± 2.5 | 0.10 |

BDR, bronchodilator response; BMI, body mass index; mMRC, modified Medical Research Council; CAT score, COPD Assessment Test; 6MWT, 6-minute walking test; BDI, Beck depression inventory; BAI, Beck anxiety inventory; GOLD, global initiative for chronic obstructive lung disease; FEV1, forced expiratory volume in 1 second; FVC, forced vital capacity; FEF_25-75,_ Forced expiratory flow between 25% and 75%; RV, residual volume; TLC, total lung capacity; ACO, asthma-COPD overlap; IgE, immunoglobulin E; FENO, fractional exhaled nitric oxide; ICS, inhaled corticosteroid; M-S exacerbation, moderate-to-severe exacerbation; S exacerbation, severe exacerbation

Figure S1. Interactive effects of disease severity and BDR on the exacerbation rate


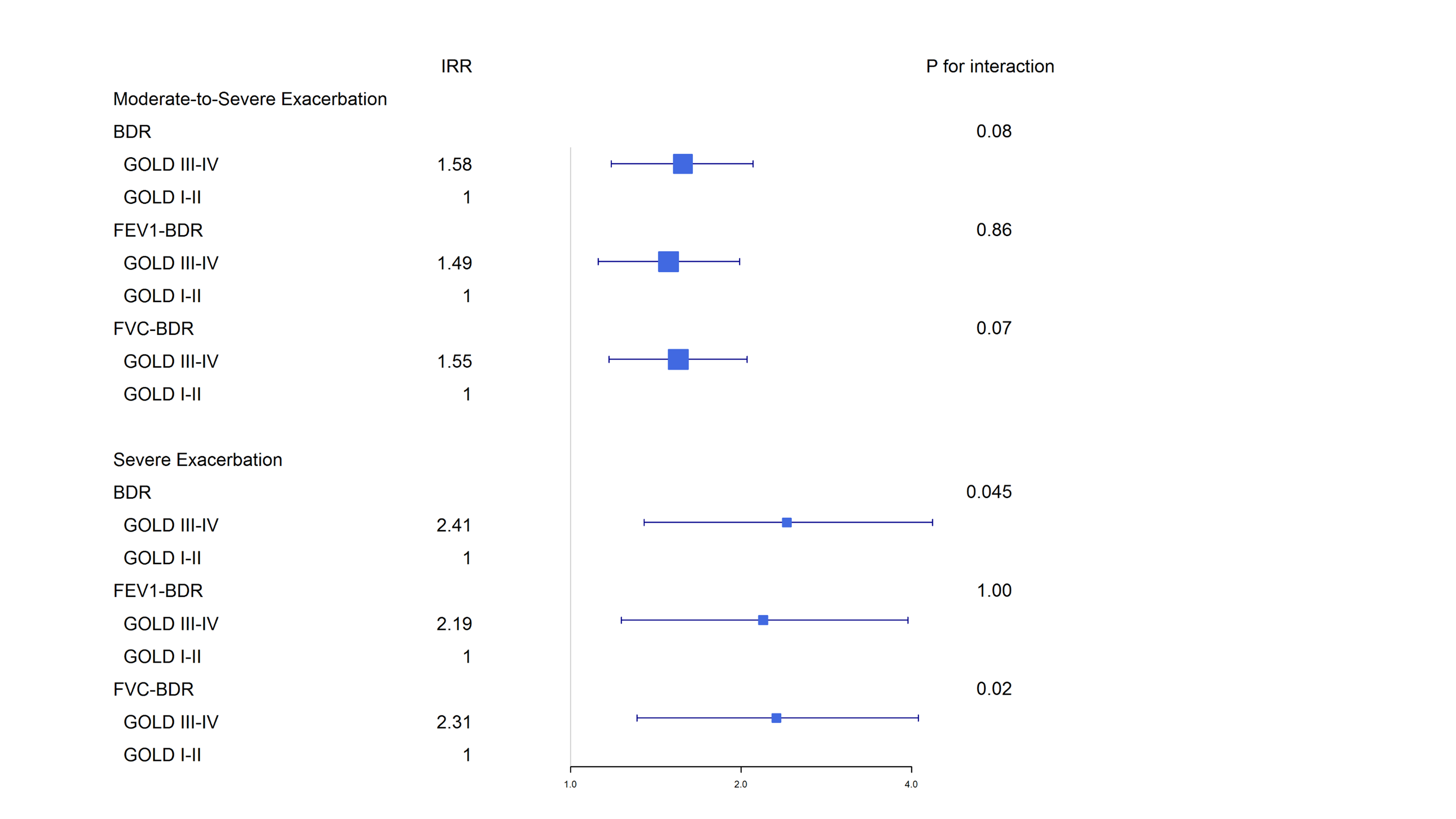

Supplement: S1 File — (DOCX) [file pone.0282256.s002.docx]
